# The Charcot shower

**DOI:** 10.1055/s-0046-1817034

**Published:** 2026-05-05

**Authors:** Marianna Selikhova, Marcelo Miranda, Andrew Lees

**Affiliations:** 1 Southmead Hospital, Bristol Brain Centre, Neurology Department, Bristol, United Kingdom.; 2 Reta Lila Weston Institute for Neurological Studies, UCL, National Hospital, Queen Square London UK.; 3 Fundación Diagnosis, Clinica MEDS, Department of Neurology, Santiago, Chile.

**Keywords:** Hydrotherapy, Jean-Martin Charcot, jet shower, Hysteria

## Abstract

Jean Martin Charcot (1825–1893) was an enthusiast of hydrotherapy for the alleviation of neurological symptoms. At the Salpêtrière hospital, he frequently advised the use of a high-pressure shower, which delivered strong pulsing jets of cold water. This therapy gained particular popularity in Russia, where it remains widely used and is known as the Charcot shower treatment.

Jean-Martin Charcot (1825–1893) is widely acknowledged as the founder of modern neurology based on his meticulous application of the anatomo-clinical diagnostic method to the study of nervous disease. Many medical eponyms are associated with his name, including two symptom triads (biliary and cerebellar), a neuropathic joint, and several neurological diseases. He is less remembered for his therapeutic innovations, including suspension treatment for locomotor ataxia, and a vibrating chair and tropane alkaloids for the treatment of Parkinson's disease. 1

Charcot formed a hydrotherapy division at his department at the Salpêtrière, where a large swimming pool resembling a Roman bath with majestic stairways was built in 1884, sometimes jokingly referred to as ‘The Temple of Balneotherapy’. 2 Some of Charcot's private patients were admitted to Dr. Fleury's Institut Hydrotherapique de Passy-Paris, a magnificent establishment located on the heights of the Trocadero, where hydrotherapy was combined with isolation from what Charcot considered to be a pathogenic family environment. 3

Based on his patients' positive reports, Charcot became particularly enthusiastic about the use of pulsating cold jets of water, which he believed improved blood circulation and indirectly stimulated the nervous system. ( Figure 1 ) The patient stood 2.5 to 3 metres away with their back facing the shower, which was then fired at a pressure of 1.0 to 3 atmospheres for a minute, using water temperatures ranging between +10 and +20 degrees Celsius. 4 This “ *douche générale au jet* ” was particularly valued for the treatment of neuropathic pain and in the rehabilitation of conditions such as multiple sclerosis, spinal cord injuries, and stroke. 1

**Figure 1:**
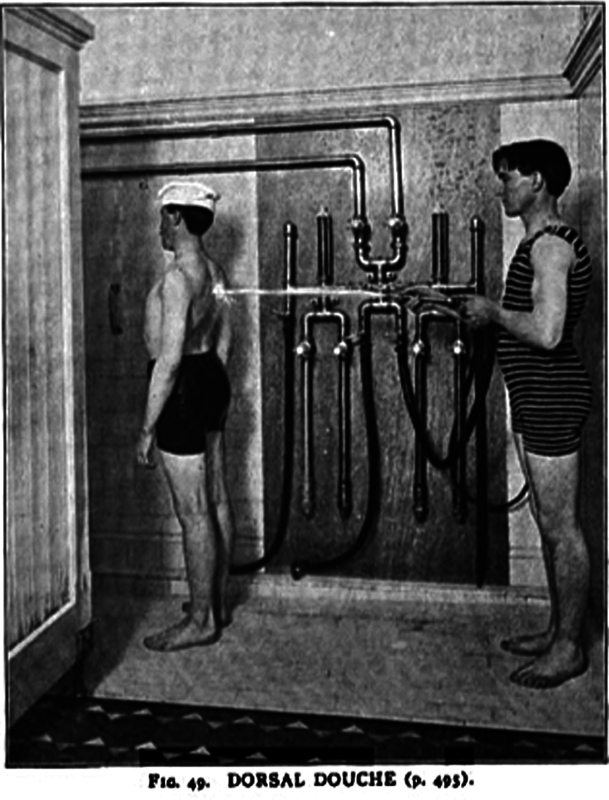
Dorsal shower in Institut Hydrothérapique de Passy-Paris. 15

In Volume 3 of *Leçons sur les maladies du système nerveux* , published in 1889, Charcot describes the successful application of the jet shower in a patient with hysteria: 5

“A young woman presented with sudden mutism. We prescribed hydrotherapy. During the first application of the jet of water, she let out a cry of surprise. Within a fortnight after the first treatment, her voice had resumed its typical character and intensity. In 1878, there was a relapse, and she again became mute. Upon the reapplication of the jet shower, Mademoiselle X eventually recovered and no further relapse over a 10-month follow-up.”

Charcot also describes in his lectures the use of the shower as beneficial in the treatment of his two celebrated male hysterics, *Porcz* and *Pin* . 5

John Harvey Kellogg (1852–1943), an American businessman, inventor of breakfast cereals, and a physician, attended Charcot's public lectures and, on his return from Paris, founded the Battle Creek Sanitarium, in Michigan. In his book *Rational Hydrotherapy* (1901), he wrote that the cold spinal shower is sometimes known as the Charcot shower, though not devised by him. 4 Kellogg was incredibly impressed with the remedial powers of water and what was referred to as “hydriatic massage” for the treatment of neurasthenia, hysteria, and other nervous maladies, but criticized its unscrupulous promotion by what he called “cold-water doctors”.

Michael Stepanovich Zernov (1857–1938), a Russian physician, attended Charcot's lectures at the Salpêtrière hospital in the 1880s and was also fascinated by the results he witnessed with hydrotherapy. Upon his return to Russia, he wrote: “I introduced in Moscow a shower of Professor Charcot, whom I met personally while visiting Paris.” 6 Zernov later also opened balneological treatment centres in Sochi and Yessentuki, which were described at the time as “a veritable paradise”, equipped with mud baths, underwater massages and jet showers. Wealthy Russians bought season tickets for the resort, and some also generously sponsored ordinary people who could not afford treatment. 6 7

Anton Chekhov (1860–1904), one of Russia's most prominent writers, who was also a physician, in his story “On Christmastide” (1900), describes a doorman named Andrei Irišanovich at the St. Petersburg sanitarium, who recommended the Charcot shower to a Russian general to relieve stress. 8

Charcot's neuroscientific work attracted great interest in Russia, and many physicians went to study under him in Paris between 1883 and 1889. These included Vladimir Mikhailovich Bekhterev, founder of the first neuropsychiatric institute in St Petersburg, Liveriy Osypovich Darkshevich, founder of the Kazan School of Neurologists, and Lazar Minor, head of the Moscow Neurological Clinic. 9 10 11 12

Many wealthy Russians also traveled to Paris to consult Charcot in his consulting rooms at the Hôtel de Varengeville. 13 An 18-year-old daughter of confectionery manufacturer Abrikosov, Sonya, who had lost the use of her legs, was diagnosed with hysteria and prescribed a course of showers by Charcot. Her nephew, Khrisanf Abrikosov, later recalled that Charcot made an extraordinary impression on Sonya. One day, she fully recovered and returned to Moscow. 14

A 19-year-old Russian nobleman and a future Minister, Paul Ignatieff, was diagnosed with hysteria and placed, according to his family's memoirs, in “Charcot's rest home in Passy” in 1889 for 6 months. This was almost certainly L'Institut Hydrothérapique de Passy-Paris that was then under the directorship of Dr Noël Pascal (1814–1889). 3 Hysteria was treated according to Dr Fleury's doctrines on rational applications of cold water with a regimen of cold wraps, baths and showers, as well as gymnastics and fencing. Pascal reported that Charcot rejuvenated the use of hydrotherapy in 1872 with his discovery of hysterogenic zones. Below is one of Pascal's observations: 15

“Miss X, 16 years old, was diagnosed by Professor Charcot with hysteria-epileptic seizures, recommended hydrotherapy in Passy and separation from the family. On admission, the patient experienced attacks of movements in an arc and incoherent speech. Upon waking, she remained incoherent for 20 minutes, then calmed down, but was still unable to walk. On initiating the shower jet, the attack reoccured, so it was switched to the filiform shower. Dr. Guilland, operating the pump, gave this shower considerable pressure, about twelve atmospheres. After several minutes of this application, the attack was under control, and the patient slept all night. On the third day, the attacks had completely ceased. After four months of treatment everything had returned to normal.”

In 1882, Pascal founded his journal on hydrotherapy *,* providing a rare insight into the symptoms and treatment of Charcot's private clientele. 3

Charcot's shower is still used, although less frequently, in hospitals and spas, mainly in Eastern Europe, Russia, post-Soviet countries, Germany, and Cuba, and is believed to enhance lymphatic drainage and alter the excitability of sensory and motor nerves. 16 It must be delivered by a specialist and details of its use are included in modern handbooks on hydrotherapy. 17 18

Two hundred years since Charcot's birth, we would like to draw the attention of neurologists to Charcot's enthusiasm for using hydrotherapy in neurological practice, and to another Charcot eponym, unfamiliar in many countries, including France. The beneficial effect of the Charcot shower may be of interest in the modern management of functional neurological disorders.
